# Thermo-Physical Characterization of Carbon Nanotube Composite Foam for Oil Recovery Applications

**DOI:** 10.3390/nano10010086

**Published:** 2020-01-02

**Authors:** Elpida Piperopoulos, Luigi Calabrese, Amani Khaskhoussi, Edoardo Proverbio, Candida Milone

**Affiliations:** 1 Department of Engineering, University of Messina, 98166 Messina, Italy; epiperopoulos@unime.it (E.P.); lcalabrese@unime.it (L.C.); eproverbio@unime.it (E.P.); 2 National Interuniversity Consortium of Materials Science and Technology (INSTM), 50121 Florence, Italy; khaskhoussiamani105@gmail.com

**Keywords:** carbon nanotubes, functionalization, silicone foam, oil recovery, sorption

## Abstract

To meet the increasing demands for effective cleanup technologies to deal with the oil spill accidents that significantly affect the ecological and environmental systems, promising composite materials based on carbon nanotubes containing silicone foams were investigated. Pump oil, kerosene, and virgin naphtha had been used to assess, during sorption tests, foams behavior. Test results highlighted the advantage of the hydrophobic and oleophilic behavior of carbon nanotubes, and their high mechanical strength for oil spill recovery application was studied. In order to better relate the property-structure relationship for this class of materials, the role and influence of functionalized nanotubes on thermo-physical and morphological characteristics of the foams had been evaluated. The results showed how the pristine nanotubes fillers, despite functionalized ones, led to optimal composite foam performances with high hydrophobic (62 mg g^−1^) and oleophilic (6830 mg g^−1^ in kerosene oil) characteristics. The evidenced high oil selectivity was a relevant key point in order to consider the suitable material for oil spill recovery applications. Eventually, the proposed configuration exhibited the best thermo-physical performances and high reusability, leading to the optimal cost-benefits option.

## 1. Introduction

Oil spill accidents all over the world, for decades, have pushed scientists to find an immediate recovery technology for the rapid removal of spilled oil from marine areas. The 20 April 2010 oil spill disaster of the Deepwater Horizon oil plant in the Gulf of Mexico is up to now considered the largest spill in U.S. waters, hiding the 1989 Exxon Valdez spill [1]. But, unluckily, numerous disasters have continued to be protagonists of bad news in our newspapers. The last one occurred in the Solomon Islands, where on 5 February 2019, the huge Hong Kong-flagged bulk carrier MV Solomon Trader, carrying 700 tons of oil, ran aground on Kongobainiu reef after becoming loose from its mooring. The oil recovery, unfortunately, remains an unsolved problem, waiting for a fast solution. Generally, there are three different methods of oil spill cleanup, i.e., mechanical [2], chemical, and biological [3]. Among these methods, the use of absorbents materials is considered the most effective approach because of their low cost, high selectivity towards oil, easy fabrication, environmental harmlessness, and recyclability. The promising materials have to possess both hydrophobic and oleophilic behavior to selectively absorb oil while repelling water. Many natural sorbents, in particular, sawdust [4], wool fiber [5], and zeolite [6] have been widely investigated for this purpose, for their high surface area and their porous structure. But most of them present low sorption capacities, poor selectivity, and no recyclability. Instead, the application of synthesized materials [7], despite the good selectivity and high sorption capacity, is still limited by expensive raw materials and complex synthesis procedures. Thus, new materials are requested. PDMS (polydimethylsiloxane) is one of the most commonly used polymers in biomedical science and material engineering [8,9]. The Si–O–Si backbone of the PDMS compound provides interesting properties to the material, i.e., non-toxicity, non-flammability, low density, and high chain flexibility. Furthermore, the intermolecular forces between the side chains of PDMS lead to low surface energy and superhydrophobicity [8]. Moreover, PDMS materials respond to a low production cost and present a high absorption capacity compared to other polymers. These properties make them exploitable for oil remediation applications. Choi et al. proposed a PDMS sponge for the selective absorption of oil from water [10]. The presented PDMS sponge was obtained by a sugar-templating process, from commercially available cube sugars (400–500 µm grain size). The sponge could be elastically deformed in any shape and compressed more times without structural collapse. Si et al. [11] controlled PDMS sponges’ morphology and enhanced their pore interconnectivity for oil sorption by partially melding sugar particles (400–1600 µm) before creating a continuous PDMS-based matrix. In order to obtain a material with reliable sorption performances, an optimal pore size distribution coupled to an improved chemical inertness, high mechanical strength, and effective water to oil selectivity is required. In such a context, carbon nanotubes (CNTs) have demonstrated to possess a good potential in the oil spill recovery applications, with a number of advantages, including stronger mechanical properties, fast sorption rates, high absorbent capacity, and well-engineered surface chemistry. Wang et al. [12] reported the design, production, and characterization of carbon nanotubes decorated with iron oxide nanoparticles for oil spill separation. The technology provided a two-steps mechanism: at first, the CNT was dispersed at the oil-water interface; afterward, they were dragged out with oil droplets by a magnet. Gui et al. [13] developed a magnetic carbon nanotube sponges, with porous structure constituted by interconnected CNT with Fe encapsulation. The CNT sponge showed high mass sorption capacity in diesel oil (56 g/g) and was characterized by a high mechanical strength that permitted the sponge squeezing by compression and, consequently, the sponge reusability. Ong et al. [14] synthesized an effective absorbent material using sugar as a sacrificial template for carbon nanotubes before the addition of PDMS compound to encapsulate CNT on its surface once polymerized. The composite sorbent presented an increase in absorption capacity, in comparison with previously reported PDMS sorbents, and a faster absorption rate. The use of composite PDMS foams filled with carbon-based fillers can be considered a promising approach in order to give a material able to selectively absorb large oil amounts from contaminated water, preserving an effective scalable and cost-effective manufacturing process of the sorbent material [15,16,17]. However, this approach has shown effective capabilities on other application contests, such as thermal or acoustic insulation systems [9]. Recently, the authors of the present article conducted a study on PDMS sponges filled with carbon nanotubes [18], obtaining a material that exhibits good oil absorbent capacities and excellent reusability. The patented process [19], described by a combined action of gas generation mechanisms and crosslinking reactions, was there illustrated. Furthermore, a relevant aspect that needs to be deeply investigated is the assessment of the relationship among morphology, surface wettability, and sorption capabilities of the CNT-based composite foam in order to acquire an improved knowledge in the property-structure characteristics on these classes of materials. In such a context, this paper focused on the fabrication and characterization of PDMS foams, reinforced with CNT filler, obtained by using a simple and low-cost process. The effects of the addition of different CNT (pristine and functionalized) on the physical properties (thermal stability, wettability, and morphology) and sorption capacity of the composite foams were discussed. The sorption capability of unfilled and filled PDMS foams was evaluated in different oil pollutants in order to assess the relationship between CNT composite foam microstructure, surface energy, and oil spill recovery efficiency. It is expected that this paper would be helpful in providing a preliminary understanding of the physical properties of CNT-filled PDMS foams to facilitate the industrial realization and application in the oil spill field.

## 2. Materials and Methods

### 2.1. CNT Synthesis

Highly purified carbon nanotubes (CNTs) were prepared by chemical vapor deposition (CVD) according to reference [20]. Fe/Al_2_O_3_ (500 mg), reduced at 500 °C in 60 cc/min H_2_ flow, was used as a catalyst. The synthesis was carried out in the i-C_4_H_10_/H_2_ (60/60 cc/min) atmosphere over 17 wt% nominal Fe load catalyst. After synthesis, raw materials were treated with NaOH solution (1 M) at 80 °C for Al_2_O_3_ support removal, while an HCl solution (37 wt%) at room temperature was used to eliminate residual Fe [21]. Synthesized CNTs were multi-walled carbon nanotubes (MWCNT), as reported by TEM analysis, here not shown for briefness. MWCNT had a mean external diameter of 14 nm and a surface area of 190 m^2^/g, measured by Brunauer–Emmett–Teller (BET) analysis. CNTs were subsequently functionalized in nitric acid vapors, as reported elsewhere [22]. The degree of chemical oxidation process was calculated by measuring the weight loss (Δw) caused by the desorption of the oxygenated species upon the inert environment in a TA Instruments SDTQ 600 (balance sensitivity: 0.1 mg). Samples (~5 mg) were heated at 20 °C/min up from 100 °C to 1000 °C using a constant flow rate of argon (100 mL/min), after preliminary sample stabilization of 30 min at 100 °C for water removal [23]. In this work, three kinds of CNT samples were investigated, pristine (pristine, p-CNT), functionalized for 2 h, with a Δw = 22 wt% (f-CNT_(22)_) at thermogravimetric analysis (TGA) and functionalized for 4 h, with a Δw = 36 wt% (f-CNT_(36)_) at TGA [18]. Moreover, in order to evaluate CNT purity and stability in the air, thermogravimetry (TG) was carried out, repeating the same conditions reported before and replacing argon flux by 100 cc/min of air. The foams filled with CNT were named hereafter, respectively, F-CNT0, F-CNT22, F-CNT36, where the number in the code was referred to the functionalization level of the CNTs. As a reference, the unfilled silicone foam was, furthermore, synthesized (F-AR).

### 2.2. Synthesis of Composite Silicone Foams

All reagents were used as supplied. Ethanol (98%) and Tin(II) 2-ethylhexanoate (Sn(II) d:1.12, M.W. 405.11, 50%, CAS. 301-10-0) were acquired from Aldrich Chemical Co. Commercially (St. Louis, MO, USA) available silicone foam reactants were purchased without any commercial filler from Gelest Inc., Morrisville, NY, USA. The system was composed of two reactants: Poly (dimethylsiloxane-co-methylhydrosiloxane) trimethylsilyl terminated (PMHS, M.W. 5500–6500 CAS: 68037-59-2) and silanol terminated polydimethylsiloxane (PDMS, M.W. 1,100,000 CAS: 70131-67-8) compounds. Tin(II) 2-ethylhexanoate was employed as a catalyst.

CNT silicone foams were prepared by a foaming approach, as reported in [18]. Initially, 0.250 g of CNT were dispersed in 2 mL of ethanol for 5 min in an ultrasonic bath and left in magnetic stirring overnight. Then, the mixture was dispersed under high shear mixing in 2 g of polydimethylsiloxane (PDMS) for about 60 s. In the meantime, the ethanol was evaporated until a constant weight was reached.

**Figure 1:**
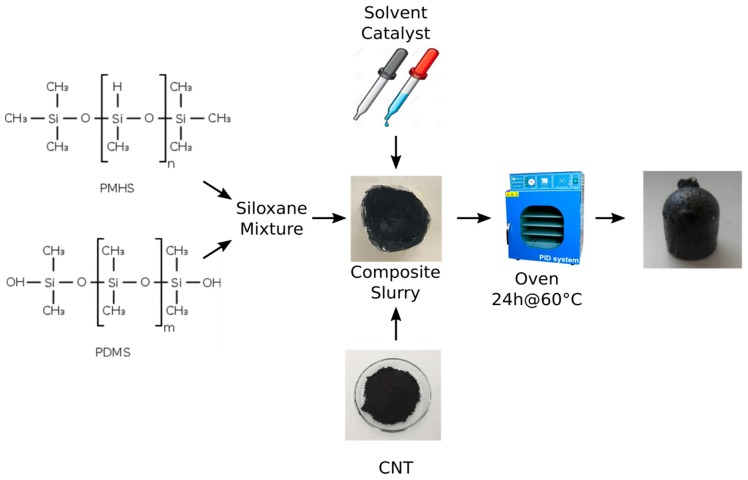
Scheme of the synthesis of the carbon nanotube composite foams.

Subsequently, 1 g of polymethylhydrosiloxane (PMHS) was carefully mixed with the CNT/PDMS mixture with a PDMS/PMHS ratio of 2:1 for 15 s. In order to reduce the composite slurry viscosity, 0.5 g of water and 0.2 g of ethanol were used as a solvent. Lastly, 0.5 g of tin catalyst was added under strong mixing for about 15 s. The final mixture was transferred into a 2 cm diameter cylindrical mold to define a proper shape during the foaming process. The foaming was obtained, thanks to the chemical reaction between PDMS and PMHS that involves the formation of hydrogen gas. To obtain the whole foaming and compound reaction, soft curing at 60 °C for 24 h was carried out in an oven. The foaming phase was activated. Different foams containing 5.6% by weight of functionalized or pristine CNT in PDMS/PMHS matrix were synthesized. A scheme of the synthesis procedure is shown in Figure 1. Details of synthesis procedure are reported in [18].

In order to study foam morphology, scanning electron microscope (FEI Quanta FEG 450) operating at 5 kV in a low vacuum and 3D optical microscopy (KH8700 3D digital microscope, Hirox) were used. The bulk (apparent) density of the foams was calculated as a weight to volume ratio.

XRD patterns were collected by a Bruker D8 Advance instrument, in the range 10–80° with a step of 0.1°/s, with a Bragg–Brentano theta-2theta configuration and a Cu Kα radiation (40 kV, 40 mA).

### 2.3. Sample Characterization

#### 2.3.1. Evaluation of Sorption Capacity

Three commonly used oils (kerosene, virgin naphtha, and pump oil) were employed for the sorption experiments. The density (ρ), dynamic viscosity (µ), and surface tension of the investigated oils are summarized in Table 1.

**Figure 2:**
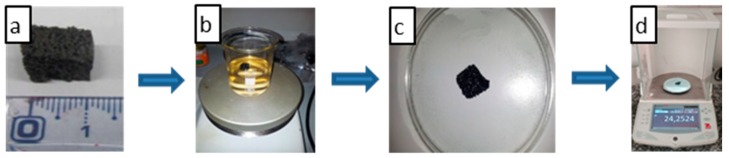
Sorption test procedure: The investigated foam (**a**) was dipped in the selected oil (**b**) at room temperature, under slow stirring, then the foam was left to rest on watch glass (**c**) for 30 s in order to remove the residual liquid and weighed (**d**).

The investigated foams of about 1 × 1 × 1 cm^3^ volume (Figure 2a) were dipped into the selected oil (Figure 2b) at room temperature under slow stirring. The sorption experiments were carried out in 250 mL beakers, filled with 100 mL oil, where the absorbents foams were added. Once the sorption phase was completed, the foam was weighed (Figure 2d) after leaving it to rest on the watch glass for 30 s (Figure 2c) in order to remove the residual liquid.

The mass sorption capacity was calculated as:

(1) $$Q_{t}=\frac{m_{t}-m_{0}}{m_{0}}$$

where *Q_t_* is sorption capacity of the foam at *t* sorption time. *m_t_* is the weight of the sample after *t* sorption time, and *m*_0_ is the initial weight of the sample. The saturation of sorption capacity was computed when the sorption capacity did not change with time.

#### 2.3.2. Contact Angle Measurement

The static water contact angles of the foams were measured by using Attension Theta Tensiometer equipment by Biolin Scientific according to the sessile drop technique. A distilled water droplet (3 µL) was set on the sample surface at room temperature (25 °C). A micro CCD camera equipped onsite recorded the images of the droplets to be further analyzed by suitable PC Attension software (OneAttension V. 2.3) to obtain the static contact angles of different liquid droplets on each of the foams. Ten replicas of contact angle measurements for each liquid (water, pump oil, kerosene, naphtha), located on regular surface areas of composite foams, were performed.

#### 2.3.3. Thermogravimetric Analysis

Thermal stability in the air of investigated foams was evaluated by a thermogravimetric analysis conducted with TA Instruments SDTQ 600 (balance sensitivity: 0.1 mg). Samples (~15 mg) were heated at 20 °C/min from 100 °C up to 1000 °C using a constant airflow rate (100 mL/min), after preliminary sample stabilization for 30 min at 100 °C to remove the eventually adsorbed water. Weight loss (%) and derivative weight loss (%/min) were calculated.

## 3. Results and Discussion

### 3.1. Thermogravimetry

**Figure 3:**
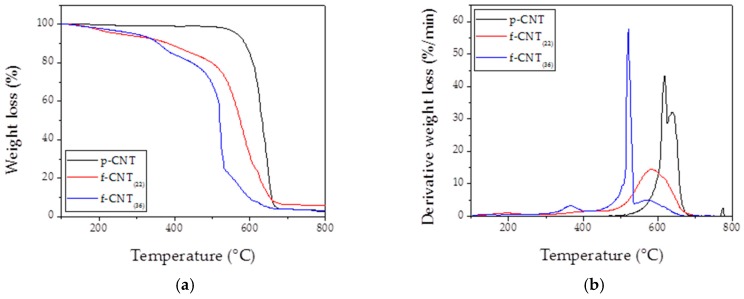
TG (thermogravimetry) (**a**) and DTG (derivative thermogravimetry) (**b**) analysis on used carbon nanotubes fillers.

In Figure 3a, TG results carried out in the air on different functionalized CNT fillers are reported. TG analysis revealed that on comparing the three kinds of used CNT, depending on their functionalization degree, the curve trends were significantly different. All the curves reached almost 0 wt% of residue, showing a low inclusion of Fe-catalyst inside the tubes [20]. In particular, the functionalized CNT showed a weight loss in the range 200–400 °C, followed by a considerable weight loss (~80 wt%) in the range 400–700 °C. Preliminary tests evidenced that amorphous carbon and oxidized groups burned in the range of 200–450 °C [20]. In fact, oxidized groups, like defects, led to oxidative instability by providing edges and hanging bonds by which each nanotube layer could be opened and peeled away, resulting in combustion at a temperature lower than that one of crystalline graphite structures. When external layers were removed, the process was reactivated because of new defect sites that were triggered and exposed [24]. This evidenced that the oxidized nanotubes were considerably less stable than pristine ones that showed a net mass loss, burning in the 550–700 °C temperature range. Figure 3b clearly confirms what is reported in Figure 3a. In particular, on increasing functionalization degree, the air stability of CNT decreased, moving the onset temperature of combustion and the T_max_ (temperature at which the rate of combustion is highest) at lower values (the stability rank is pristine CNT > functionalized CNT 22% > functionalized CNT 36%, with a combustion temperature of 620 °C > 580 °C > 520 °C, respectively). Based on these observations, we conjectured that pristine nanotubes exhibited greater oxidative stability.

**Figure 4:**
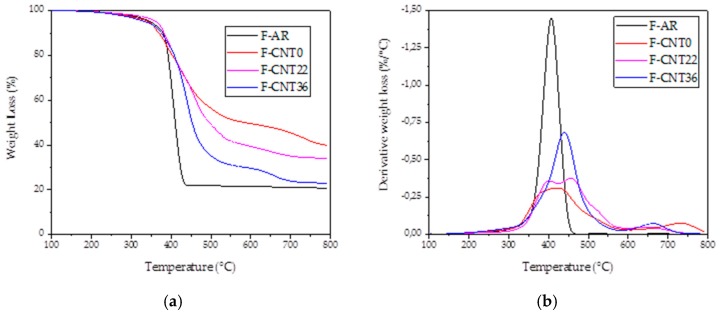
TG (**a**) and DTG (**b**) analysis on investigated foams.

In Figure 4a, the TG analyses of investigated foams are shown. As presented, all the foams burned, leaving a residue around 20–40 wt%.

The main residue was constituted by the oxidated Sn due to the employed catalyst (Figure 4). Sn (II) octoate (molecular weight: 405.12 g/mol) catalyst was the 11.6 wt% in the foam formulation, as reported in Table 1. During the calcination in air, Sn oxidates according to the reaction:

Sn + O_2_ → SnO_2_ (2)

**Figure 5:**
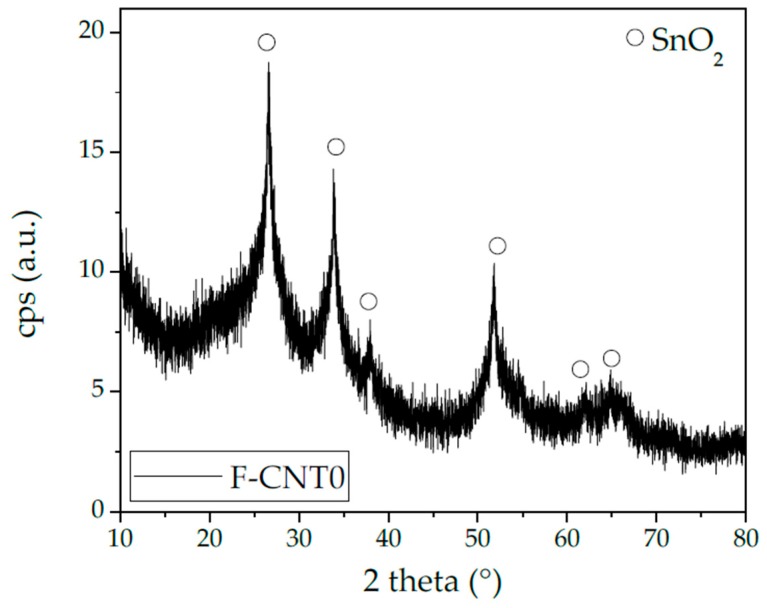
XRD analysis of F-CNT0 TGA (thermos gravimetric analysis) residue.

The SnO_2_ (molecular weight: 150.71 g/mol) became the 5.6 wt% of the foam’s non-volatile components (PDMS, PMHS, filler, and SnO_2_). The remaining part of the residue was probably due to the SiO_2_ phase because of the oxidation of PDMS in the air condition [25]. Figure 5 shows the X-ray diffractogram of the F-CNT0 sample as reference (the other samples’ diffractograms were not reported here for the sake of brevity). It confirmed that both the SnO_2_ oxide peaks and the presence of an amorphous phase (probably amorphous SiO_2_) could not be excluded.

Moreover, the T_max_ at which the combustion temperature took place is 408 °C (Figure 4b). If CNT fillers were added in PDMS foam, a different behavior appeared. Two main weight loss steps were evident. The first step (in the range of 350–500 °C) was related to a relevant weight loss, and it was mainly ascribed to the PDMS combustion. This amount increased, increasing the functionalization degree of CNT. The rank was F-CNT0 < F-CNT22 < F-CNT36, with weight loss of 38.3% < 50.6% < 65%, respectively. Increasing the temperature up to 600 °C, a further weight loss gap took place. This second step was less relevant and probably was due to an increase in the rigidity of the siloxane chains [26]. The residues of the CNT filled foams were 47.4% for F-CNT0, 34.1% for F-CNT22, and 22.9% for F-CNT36. These results indicated that the thermally stable CNT network offered a transient protective barrier action in the nanocomposite material [26]. This limited the depolymerization of the siloxane chains. The residues of CNT foams appeared as grey cracked foams that easily shattered to touch. In all the cases, considering that the oil absorption experiment usually is performed at room temperature, which is no more than 100 °C, the sponges were stable under real service conditions.

In addition, CNT fillers increased the foam combustion temperature, as reported in Figure 4b, enhancing the thermal stability of PDMS foam. For F-CNT0 and F-CNT22, the combustion peaks appeared wider because the lower residue and the concomitance of more combustion phases, and consequently, T_max_, were not clearly identifiable. For F-CNT36, the displacement at a higher peak temperature (441.2 °C) was visibly detectable (inset in Figure 2b). This behavior has previously been explained by different mechanisms: (i) dispersed carbon nanotubes may obstruct the degradation flux products; (ii) polymers chains may destroy more slowly near the nanotubes, shifting T_max_ to higher temperatures; (iii) higher thermal conductivity in the composites, induced by nanotubes presence may promote heat dissipation in the composite [26]. Furthermore, the presence of CNT, as reported by Kim [27], could lead to the stabilization of PDMS matrix interlayer, inducing protective barriers against thermal decomposition, and retarding the thermal decomposition of PDMS. So, it could be confirmed that the presence of CNT enhanced the thermal stability of PMDS composites foams. Also, the functionalization of nanotubes enhanced the thermal stability of composites. This behavior could be ascribed to the surface functional groups in the CNT. In fact, the hydrogen bonding interaction and good interfacial adhesion between the functionalized CNT and PDMS matrix restricted the thermal motion of PDMS macromolecules, leading to a further increase in their thermal stability [28].

### 3.2. Morphology

**Figure 6:**
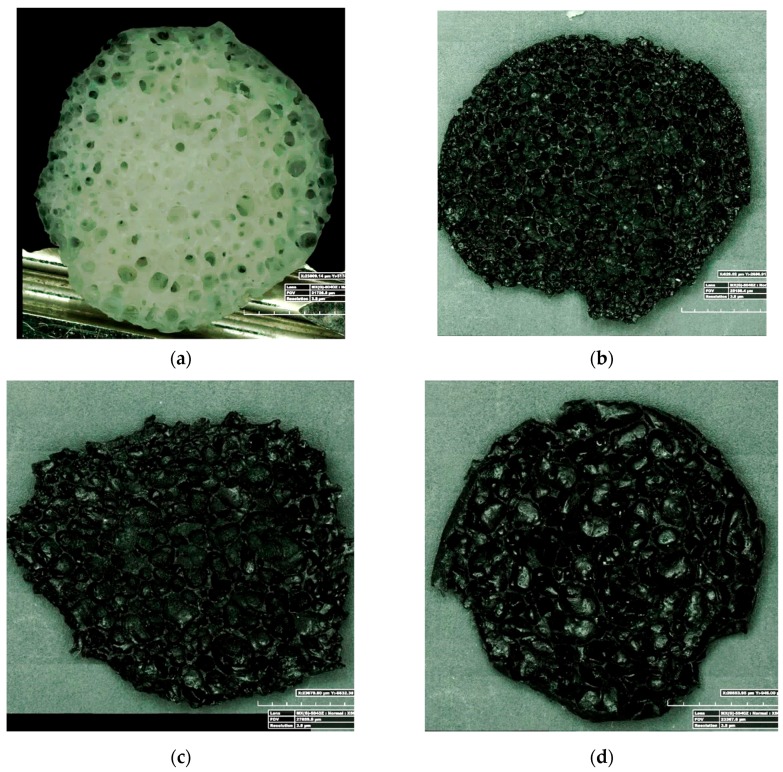
Cross-section optical images of (**a**) F-AR, (**b**) F-CNT0, (**c**) F-CNT22, and (**d**) F-CNT36 foams.

In Figure 6, 3D optical micrographs of the cross-section for all foam samples are reported. All foams showed a porous isotropic structure without significant heterogeneities or preferential orientation. The bubbles were randomly distributed and interconnected with each other. The porous structure morphology was influenced by CNT filler addition, as identifiable comparing the micrographs of Figure 6. Mean pore size (MPS) was calculated as the average equivalent diameter of the bubble distribution, determined by SEM micrographs by software image analysis (ImageJ). The 2D pore size distribution was corrected based on to Schwartz–Saltykov (SS) criterion [29]. This mathematical approach estimated the 3D pore size distribution from a 2D pore size one, avoiding constraints concerning the form of the particle size distribution [30]. The AD of the foams was calculated as the mass to 1 cm^3^ foam sample. For each batch, five replicas were carried out.

**Figure 7:**
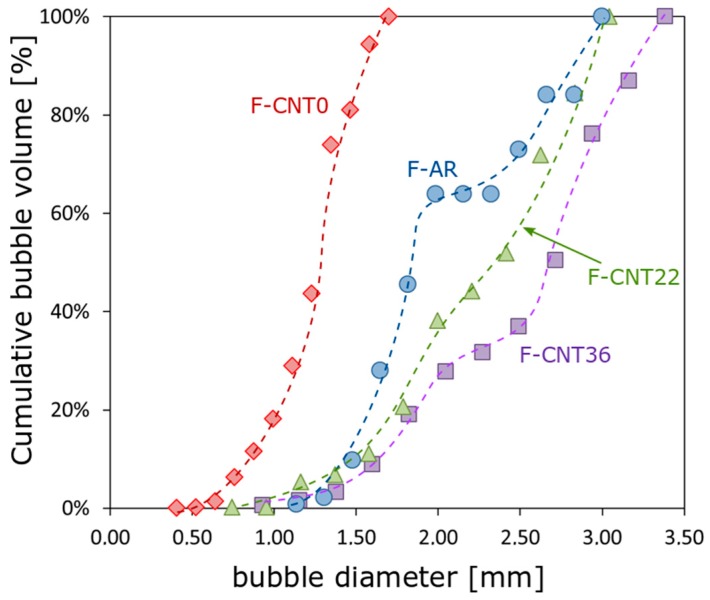
Cumulative bubble volume evolution at varying diameter for all composite foams.

Figure 7 shows the cumulative bubble volume percentage evolution at varying diameter for all composite foams.

Unfilled and functionalized CNT filled foams (F-AR, F-CNT22 and F-CNT36) showed a quite similar trend. For these samples, the cumulative area curve started at a larger bubble size dimension than F-CNT0.

The unfilled silicone foam, F-AR sample, was characterized by circular-shaped bubbles with a cumulative volume curve that started at a smaller bubble size dimension than CNT-based foam.

The curve showed a sigmoidal shape. The first section was mainly related to small size bubbles, not coalesced. Beyond a plateau, the intermediate diameter size (in the range 1.75–2.25 mm) in the trend of the curve was identifiable. Afterward, the region with a cumulative volume in the 70%–100% range was associated with a bubble size of about 2.5–3 mm. This stage could be associated with the initiation and evolution of coalescence phenomena. In this region, a relevant modification of the curve slope was evident. This behavior was related to the formation of an interconnected local structure generated because of the coalescence of aggregate bubble domains.

This sample evidenced an average diameter of about 1.75 mm and an apparent density of 280.3 kg/m^3^. The addition of pristine carbon nanotubes, F-CNT0 sample, caused a significant reduction of the bubble size (average diameter ~1.05 mm). This was confirmed by the large shift of the cumulative value curve toward a very small bubble diameter. Afterward, increasing the CNT functionalization, instead, a progressive increase of bubble size could be identified by the shift on the right of the curves. Indeed, F-CNT22 and F-CNT36 showed an average bubble size of 1.82 mm and 2 mm, respectively. These results indicated that the carbon nanotube functionalization influenced the foaming stage. The presence of functional groups on the CNT surface favored the interaction with the hydroxyl and hydride groups of silicone compounds, stimulating the formation of chemical bonds between the filler and the matrix [31]. At the same time, water and hydrogen were generated as reaction products [32], thus favoring an increase of the foaming ratio. In fact, the apparent foam density, determining the mass to volume ratio, decreased with increasing the pore size. F-AR foam presented a reference density of 280.3 kg/m^3^. The composite F-CNT0 foam, characterized by the smallest pore size, evidenced a density of 314.6 kg/m^3^ that was about 12% higher than the unfilled silicone foam, indicating a dense and compact porous structure. Due to the functionalized CNT filler, the apparent density of the composite foam decreased to 252.9 kg/m^3^ and 251.3 kg/m^3^ for F-CNT22 and F-CNT36 foams, respectively.

### 3.3. Sorption Capacity

**Figure 8:**
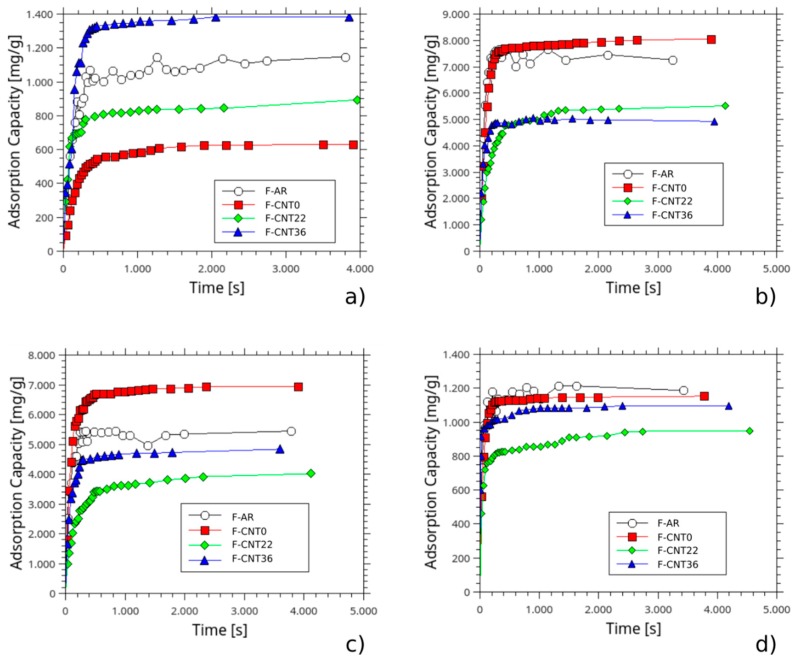
Sorption capacity at increasing sorption time for all foams in (**a**) water, (**b**) naphtha, (**c**) kerosene, (**d**) pump oil.

The absorption capacity, Q_t_ (mg/g), at increasing time for the foams in several liquids is reported in Figure 8. At first, useful information could be acquired, analyzing the absorption capacity of the silicone foams in water (Figure 8a). All batches evidenced a progressive increase of Q_t_ at increasing absorption time. A similar trend could be highlighted, analyzing the sorption capacity of the silicone foams in all other liquids (Figure 8b–d).

In order to have effective oil recovery capacity, the sorbent materials need to be able to absorb a high amount of oil pollutants but low water amounts. This characteristic is required in order to offer suitable selective sorption of pollutants in water.

Very high sorption capacity in water was observed for the F-AR sample. Instead, composite foam filled with pristine CNT, F-CNT0, provided a low interaction with the water, obtaining a maximum sorption capacity of about 53% lower than the F-AR sample. The carbon nanotubes’ functionalization induced a progressive increase of maximum water sorption at equilibrium. The highest water sorption was observed for the F-CNT36 sample. This behavior was probably related to a higher amount of polar active groups on the filler surface that could interact with water. However, as observed by Chen et al. [33], the water could also slightly interact with the siloxane matrix, as provided by intrusion pressure (P_int_, that indicates the maximum water pressure that the foam interface can suffer before the water penetrates the siloxane surface) measurements.

Analyzing the sorption capacity of composite foams by varying oil pollutants, it is worthy of note that high sorption capacity was observed in naphtha and kerosene pollutants. Sorption capacity was enhanced for low-density oils against denser ones. For the F-AR sample, pump oil sorption capacity was quite similar to water. Particularly, in pump oil pollutants (featured by a density of 858 kg/cm^3^ and a viscosity of 0.1231 Pa·s), the silicone sponge reached the saturation equilibrium after about 120 s absorbing 1120 mg g^−1^ of oil. The equilibrium time (when the sorption does not change anymore) was perceptively reduced for low-density oils because the pollutant could simply enter through the foam micro-porous structure. For kerosene (featured by a density, ρ and viscosity, µ, of 780 kg/cm^3^ and 0.0019 Pa·s, respectively), the sorption achieved the equilibrium after 240 s, sorbing 5500 mg g^−1^ of oil. Instead, higher was the density of the oils (pump oil), the lower were the sponges’ sorption capacities. The low affinity with high-density oils could be associated with the high dynamic viscosity of heavier oils (see Table 1) and to their higher surface tension. 

However, the absorption capacities of foams in water, although its low viscosity, were low. This indicated a greater affinity of the foam with these oils compared to water. The hydrophobic nature of PDMS influenced the absorption kinetics of the foam and its absorption capacity at equilibrium, limiting the active sites to interact with polar liquids, such as water, and favoring the interaction with apolar liquids, such as oil. The CNT filler was embedded in the silicone foams, exalting the hydrophobic and oleophilic behavior of the foam surface. In fact, as shown in Figure 8, CNT-F0 presented the lowest sorption value in water (62 mg g^−1^), exalting PDMS hydrophobic behavior, in comparison with the F-AR sample (1150 mg g^−1^). At the same time, CNT filler increased the PDMS oil affinity, in particular, in kerosene oil and in naphtha oil, in which the absorption capacity reached 6830 mg g^−1^ and 8000 mg g^−1^ respectively. These results were comparable and, in some cases, above the values reported in the literature. For example, Choi et al. [10] realized a PDMS sponge that approximately showed an absorption capacity in the range of 4000 to 11,000 mg g^−1^ for various oil and organic solvents, and the achieved outcomes reported in this work were almost in the same range. Moreover, Zhao et al. [Improvement of oil adsorption performance by a sponge-like natural vermiculite-carbon nanotube hybrid] for their sponge-like natural vermiculite-carbon nanotube hybrid obtained 2600 mg g^−1^ of absorption after 5 min of CNT growth on Fe/Mo vermiculite-based catalyst in diesel oil that was quite lower than our result. They reached the highest oil absorption (15,000 mg g^−1^), increasing the CNT growth duration up to 120 min. The obtained material was fluffy, and the reuse was difficult; conversely, CNT-F0 foam was able to be used several times [18]. The improvement was less visible for pump oil. In fact, as described before, the lower sorption capacity in higher density oils could be associated with the high dynamic viscosity of heavier oils, accentuated by the micro-roughness of the CNT-filled foam surface, as described elsewhere [34].

What is noteworthy is that filling PDMS foam with functionalized CNT increased the hydrophilicity of foams (820 mg g^−1^ and 1350 mg g^−1^ for F-CNT22 ad F-CNT36, respectively) and decreased the oleophilicity. The hydrophilic behavior was associated with a larger amount of carboxyl groups in the CNT surface that facilitate water sorption in the composite foam.

### 3.4. Wettability

**Figure 9:**
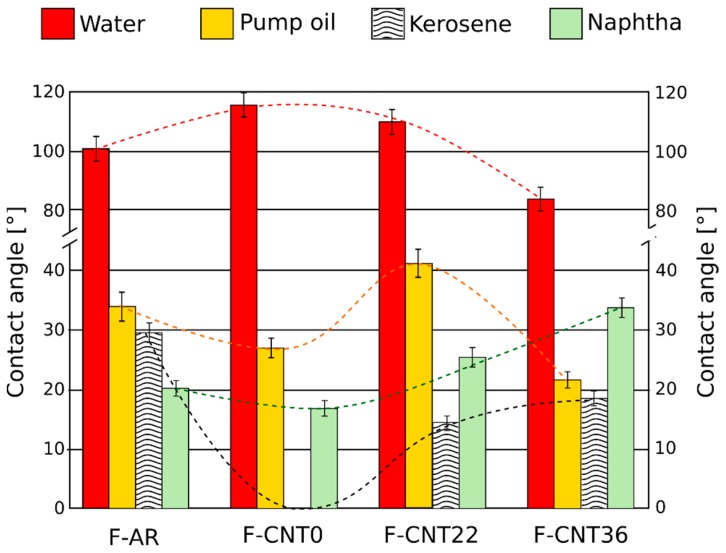
The contact angle of different liquids for all foams.

**Figure 10:**
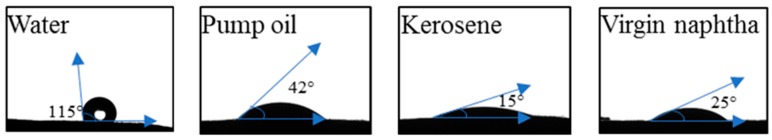
The contact angle of investigated liquids for F-CNT22.

The variation of contact angle values of all composite foam surfaces at varying pollutants represented an effective approach to identify the surface interaction of the silicone foams with the different liquids in order to relate it with the sorption performances of the foam for oil recovery (Figure 9). Furthermore, as a reference, contact angles of all investigated liquid pollutants for F-CNT22 are shown in Figure 10.

A relevant difference between contact angle in water (WCA) and the ones in pollutants (pump oil, kerosene, and virgin naphtha) could be identified. Generally, the wettability of a solid surface depends on two factors: its topographical microstructure and its surface chemical composition [1]. For all the investigated foams, the contact angle of the water was always above 70°. The unfilled foam (F-AR) had a WCA of 100.6°, above the hydrophobicity threshold, defined at 90°. The addition of carbon nanotubes enhanced the hydrophobic behavior of the surface, with an increase in the contact angle—115.9° and 110° for the foams F-CNT0 and F-CNT22, respectively. Combining PDMS with the micro-scale roughness of the hydrophobic surface of a CNT-filled foam provided us with a hydrophobic sponge [1]. However, the addition of carboxyl and hydroxyl functional groups on the surface of the nanotubes, due to functionalization, favored the interaction of the surface with water, as identifiable by a slight reduction of WCA value (about 5%) from the pristine nanotubes (F-CNT0) to nanotubes functionalized to 22% (F-CNT22). Afterward, the use of highly functionalized carbon nanotubes, F-CNT36, led to a significant reduction in the contact angle, observing an average value of 82.8° related to a hydrophilic behavior for this surface. Thus, it could be ascribed to the presence of many functional groups on the surface of the CNT that favor the surface interaction with a polar liquid, such as water. This behavior represented an obstacle to the selectivity of the absorbent foam for oil recovery, since, in order to maximize the performance of the foams for such applications, the hydrophobicity and the oleophilicity of the surface need to be enhanced.

**Figure 11:**
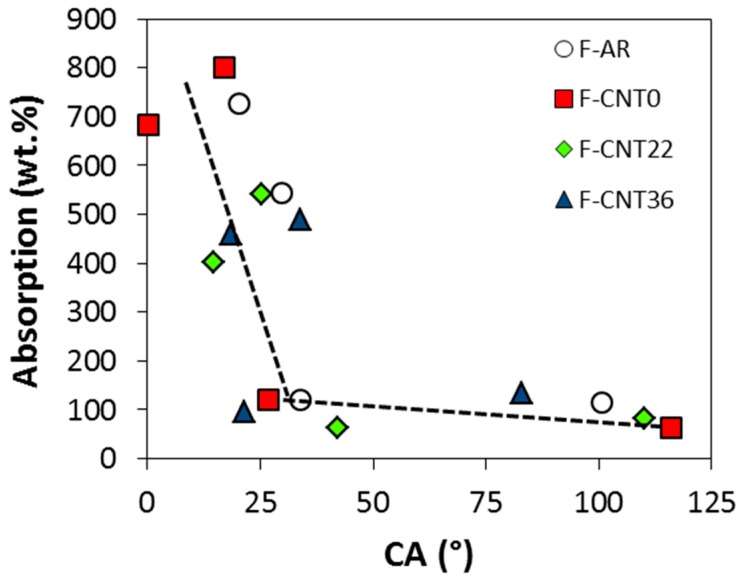
Correlation absorption (wt%) vs. contact angle (CA—(°)).

These considerations could be explained, considering the evolution of liquid sorption versus contact angle (Figure 11). It is possible to highlight that the sorption capability of the material was strictly related to the interaction of the surface with the chosen liquid. In particular, when a high contact angle of the liquid occurred, a low sorption capacity could be observed. Instead, low contact angles identified an effective interaction of the surface with the liquid; therefore, a good sorption capacity was obtained. In particular, a bilinear trend could be highlighted with a threshold value at about 30° that discriminates the high to low sorption areas of the plot.

Consequently, in order to have a good sorption capability with oils, the foams must show very low contact angles with the different pollutants in order to support liquid permeation in the interconnected porous channels of the composite foam. It was noted that nanotube-based composite foams applied effectively in this context. The lower contact angles had been identified for the F-CNT0 foam for which there was a contact angle in kerosene (KCA) equal to 0°. This was in accordance with what has been discussed about the high kerosene absorption capacity of the nanotube-based composite foam, in particular for the F-CNT0 sample. Similarly, very low contact angle values for virgin naphtha (NCA) and pump oil (PCA) were detected. However, it is useful to point out that the pump oil sorption values, as observed in Figure 8, were very low, although a relevant oleophilic behavior for all the composite foams with this pollutant was clearly identifiable. The PCA varied between 21° and 42°, highlighting the superficial interaction between the liquid and the foam. The observed trend was in agreement with sorption capacity results. Highest PCA and lowest sorption capacity were observed for the F-CNT22 sample. The images related to its wettability are reported, as an example, in Figure 10. However, although the contact angle among kerosene, naphtha, and pump oil was quite similar, a very different sorption capacity was observed with pump oil liquid (about five times lower than the other two pollutants). This behavior could be ascribed to the high viscosity of the pump oil, compared with the other pollutants, which can severely limit the mass transport phenomena within the foam porosity, with subsequent reduction of the absorption capacity of the foam itself.

In fact, the absorption phenomenon could be identified by several stages. In the first phase, at low absorption times (in the range 0–200 s), a rapid absorption phenomenon occurred that could be associated with an external mass transfer (transport from the bulk solution to the sorbent surface) of the pollutant coupled to intraparticle diffusion (pore and surface diffusion). In the second phase, occurring at medium time (in the range 200–2500 s), the absorption progressively decreased, and a plateau was reached. This sorption stage could be ascribed to two competing mechanisms: film diffusion (diffusion across the liquid film from the sorbent surface) and the surface interactions on the active sites.

**Figure 12:**
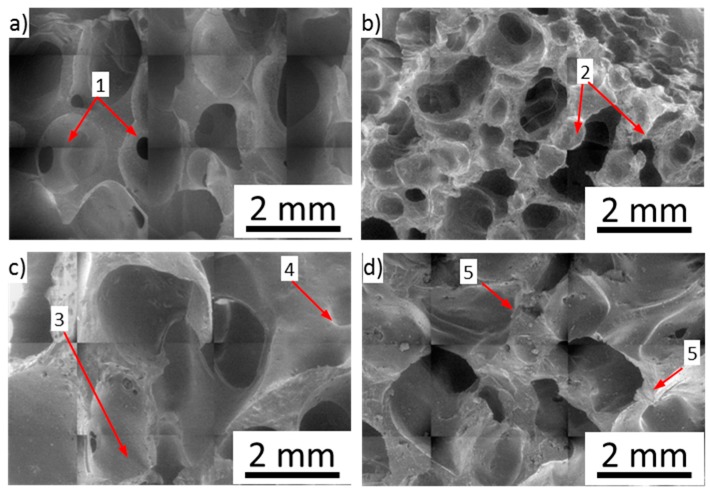
SEM images of (**a**) F-AR, (**b**) F-CNT0, (**c**) F-CNT22, and (**d**) F-CNT36 foams.

Following a preliminary rapid absorption during the first phase due to the sorption of the pollutant through the interconnected channels of the foam, the absorption in this phase could be due to the diffusion of the liquid towards the innermost micropores of the foam [35]. In such a context, the CNT filler influences effectively the oleophilic behavior of composite foam surface, increasing the surface sites able to interact with the oil, thanks to a reduced polar and dispersive surface energy [36]. This phenomenon is favored by micro-rough pore walls in the foam surface that exalt the sorption capacity of the foam. As shown in Figure 12, CNT foam walls were characterized by a rougher surface than the F-AR foam sample due to the CNT filler embedded in the silicone matrix. The surface asperities of the composite foam act as active sites for oil sorption [37].

All composite foams showed regular and almost isotropic structures (Figure 12).

The unfilled silicone foam was constituted by spheroidal shaped bubbles interconnected with each other by micro-channels and pores (point 1 in Figure 12a). The composite silicone foam filled with pristine CNT, F-CNT0, (Figure 12b) had a quite different morphology induced by the nanosize filler addition. A reduction in bubble size caused to both high viscosity of the slurry and bubble triggering effects caused by CNT addition [38] could be identified. As a consequence, non-spherical shaped bubbles due to aggregation phenomena of neighboring bubbles, which led to the generation of porosity with non-regular geometries, could be found (point 2 in Figure 12b) [39]. The bubbles were still strongly interconnected, and a three-dimensional channel branching occurred. This is a fundamental condition in order to maximize the mass transport properties through the foam channels during the first stages of absorption [40].

Analyzing composite foams with functionalized CNT fillers (F-CNT22 and F-CNT36) (Figure 12c,d), the bubble distribution was less regular with separated colonies of large or small bubbles (point 3 and point 4, respectively, in Figure 12c). This discrepancy, compared to pristine CNT foam, could be ascribed to the foaming process. During the foam synthesis, the cell size was influenced by the reaction among matrix constituents and CNT filler, influencing the chemical and physical bubbling phenomena [26]. This resulted in a more effective mechanical stability at the filler-matrix interface. Moreover, the greater chemical reactivity among the constituents involved a higher internal pressure in the formed bubbles due to the hydrogen evolution in the siloxane matrix, with consequent expansion of the bubbles themselves.

However, in F-CNT36 foam, the high surface functionalization of the nanotubes involved a high number of cross-linking points that led to a more rigid and brittle to touch composite foam. In particular, the intrinsic flexibility and compressibility of the silicone matrix were strongly limited, and the triggering of surface cracks or defects took place (point 5 in Figure 12d). Thus, it was a prelude of a less effective mechanical stability of this foam batch. Further studies would be focused, specifically addressed to evaluate the mechanical performances of foams under static and cyclic compression test in order to assess mechanical stability and durability of the composite foams.

## 4. Conclusions

In this work, porous PDMS foams filled with CNT were synthesized and characterized. CNT filler was selected to increase hydrophobicity and oleophilicity of PDMS foams. The exploitation of the composite foams as oil absorbents was conducted. The comparison between pristine and functionalized CNT was investigated on the absorption capacity, morphological, and thermo-physical characteristics of the composite foams. Functionalized CNT decreased absorption capacity and thermal stability, increasing mean pore size and decreasing foam bulk density. Pristine CNT enhanced the properties of the PDMS foam, presenting high absorption capacity (6830 mg g^−1^ in kerosene oil), good thermal stability (residue = 47.4 wt%), and hydrophobicity (WCA: 118°), making this material appealing for low-cost high-throughput fabrication of a novel family of oil recovery sorbents.

## Figures and Tables

**Table 1:** Density (ρ), dynamic viscosity (µ), and surface tension of selected oils.

	Density, Ρ (kg/m^3^)	Dynamic Viscosity μ (Pa·s)	Surface Tension (in air @ 25 °C) (10^−3^ N/m)
Water	1000	0.00100	71–79
Kerosene	780	0.0019	23–32
Virgin Naphtha	630	0.0012	18–26
Pump Oil	858	0.1231	27–35
